# The N-terminal fragment of the tomato torrado virus RNA1-encoded polyprotein induces a hypersensitive response (HR)-like reaction in *Nicotiana benthamiana*

**DOI:** 10.1007/s00705-016-2841-8

**Published:** 2016-04-13

**Authors:** Przemysław Wieczorek, Aleksandra Obrępalska-Stęplowska

**Affiliations:** Interdepartmental Laboratory of Molecular Biology, Institute of Plant Protection-National Research Institute, 20 Władysława Węgorka St, 60-318 Poznan, Poland

## Abstract

The hypersensitive response (HR) is a defence reaction observed during incompatible plant-pathogen interactions in plants infected with a wide range of fungi, bacteria and viruses. Here, we show that an N-terminal polyprotein fragment encoded by tomato torrado virus RNA1, located between the first ATG codon and the protease cofactor (ProCo) motif, induces an HR-like reaction in *Nicotiana benthamiana*. *Agrobacterium tumefaciens*-mediated transient expression of the first 105 amino acids (the calculated molecular weight of the fragment was ca. 11.33 kDa, hereafter refered to as the 11K domain) from ToTV RNA1 induced an HR-like phenotype in infiltrated leaves. To investigate whether the 11K domain could influence the virulence and pathogenicity of a recombinant virus, we created a potato virus X (PVX) with the 11K coding sequence inserted under a duplicated coat protein promoter. We found that 11K substantially increased the virulence of the recombinant virus. Disease phenotype induced in *N. benthamiana* by PVX-11K was characterized by strong local and systemic necrosis. This was not observed when the 11K domain was expressed from PVX in an antisense orientation. Further analyses revealed that the 11K domain could not suppress posttranscriptional gene silencing (PTGS) of green fluorescent protein (GFP) in the *N. benthamiana* 16c line. *In silico* analysis of the predicted secondary structure of the 11K domain indicated the presence of two putative helices that are highly conserved in tomato-infecting representatives of the genus *Torradovirus*.

## Introduction

Interactions between plants and pathogens represent an everlasting arms race between the invader and the host. These interactions also take place at the molecular level, involving nucleic acids and proteins of the pathogens (fungi, bacteria, viruses) as well as those of the plant. Viruses take advantage of the cellular machinery to replicate and spread within the host. This reprograms cell metabolism and leads to a sequence of events that negatively affect the development of the invaded plant.

Some viruses induce systemic necrosis in the plant upon infection [1]. In contrast to a hypersensitive reaction (HR), which limits the spread of the virus to a local focus of infection, systemic necrosis does not prevent the virus from further spreading within the infected host [2]. Importantly, systemic necrosis has been proposed to share some biochemical and physiological features with programmed cell death (PCD) [3].

**Fig. 1:**
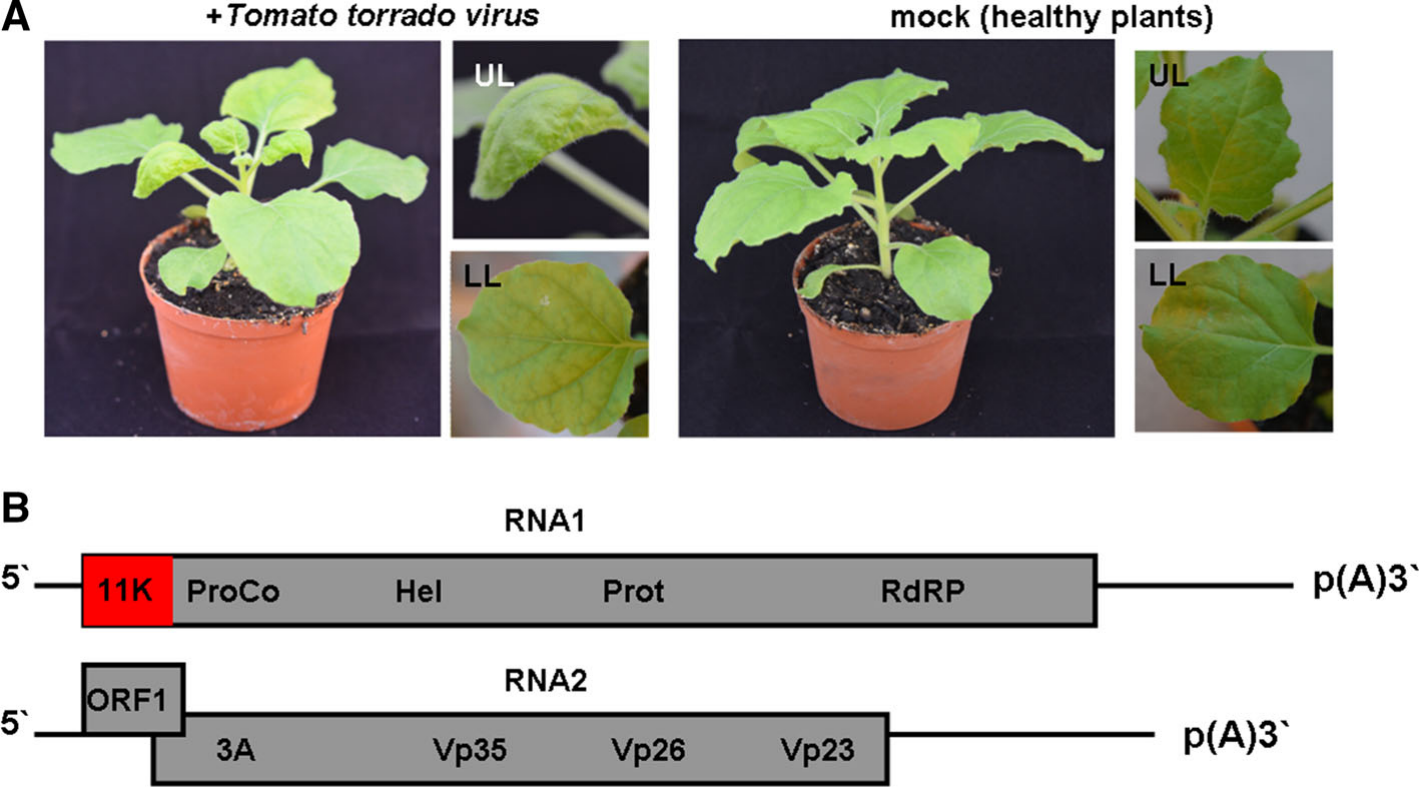
**A.** Symptoms induced in *Nicotiana benthamiana* plants upon tomato torrado virus (ToTV) infection. Healthy (mock-inoculated) plants were included. UL and LL, magnification of upper and lower leaves, respectively. **B.** Schematic representation of the ToTV genome. The two RNA strands are shown. RNA1 encodes a polyprotein with protease cofactor (ProCo), helicase (Hel), protease (Prot) and RNA-dependent RNA polymerase (RdRP) domains. RNA2 contains an open reading frame of unknown function (ORF1), and ORF2, encoding a polyprotein with a movement protein domain (3A) and three coat protein subunits (Vp35, Vp26 and Vp23). Both RNA strands are polyadenylated (pA) and flanked by 5’ and 3’ untranslated regions. The red box highlights the position of the 11K domain within the N-terminus of the RNA-encoded polyprotein (color figure online)

Tomato torrado virus (ToTV), a member of the family *Secoviridae*, is an emerging pathogen of plants of the family Solanaceae, especially tomato (*Solanum lycopersicum*) [4, 5]. The virus is considered one of the most dangerous tomato pathogens because of its virulence, wide host range and increasing worldwide distribution [6]. In tomato, ToTV induces severe systemic necrosis, which further reduces plant growth. ToTV also infects *Nicotiana benthamiana*, inducing chlorosis and leaf malformation (Fig. 1A). ToTV has an RNA genome consisting of two single-stranded (+)RNAs. RNA1 (7829 nucleotides [nt]) encodes a long polyprotein (polyprotein 1) with putative motifs involved in virus replication: protease cofactor (ProCo), helicase (Hel), protease (Prot) and RNA-dependent RNA polymerase (RdRP) (Fig. 1B). RNA2 (5404 nt) contains two open reading frames. ORF1, whose function is unknown, partially overlaps the second ORF, which encodes a long polyprotein (polyprotein 2) with a predicted movement protein motif (3A) and three ORFs for Vp35, Vp26 and Vp23 coat proteins (CP) that together form the ToTV capsid (Fig. 1B). The two long ToTV polyproteins undergo posttranslational processing in the presence of a virus-encoded protease, giving rise to the mature functional proteins [4, 5]. The precise cleavage sites within ToTV polyproteins have not yet been experimentally verified [4]. Moreover, little is known about the molecular aspects of ToTV infection in terms of virus replication or the defence mechanisms activated in ToTV-infected plants. It has been proposed, however, that virus virulence, aggressiveness and vector-mediated spreading potential might be related to variable regions (VR) residing within the 3’-untranslated region (3’UTR) of ToTV RNA1 [7] or its movement protein (3A) [8].

The transient expression approach has been widely used in preliminary studies aiming at identifying the biological function of virus-encoded proteins [9]. In the present study, we focused on analysing potential pathogenesis-inducing factors of ToTV. Using an approach based on *Agrobacterium tumefaciens*–mediated transient protein expression in *N. benthamiana*, we tested the biological effect of functional domains of the two polyproteins encoded by the virus. We performed a functional analysis of a putative 105-amino-acid protein domain residing within the N-terminal part of ToTV polyprotein 1. The putative short region, with a predicted molecular weight of 11.33 kDa (thus refered to as the 11K domain), induced an HR-like reaction when transiently expressed in *N. benthamiana*. Moreover, the 11K domain substantially enhanced the severity of symptoms produced by infection with a chimeric potato virus X-11K (PVX-11K). In many instances, the biological role of heterologously expressed viral proteins that induce necrosis in *N. benthamiana* has been correlated with their suppressive function during posttranscriptional gene silencing (PTGS) [10, 11]. However, our results did not confirm that the ToTV 11K domain could suppress PTGS. Further sequence analysis of the 11K indicated that the domain is likely to contain helical structures. The two predicted helices were found to be conserved in sequence among known tomato-infecting torradoviruses: tomato marchitez virus, tomato chocolate virus, tomato chocolate spot virus and tomato necrotic dwarf virus [12–15]. We discuss potential functions of the 11K domain in the context of its conservation among members of the genus *Torradovirus*.

## Materials and methods

### Plant material and virus source

*N. benthamiana* plants (wild type and the 16c line, expressing the green fluorescent protein [GFP]) were grown under greenhouse conditions at 24-25 °C. An infectious clone of the tomato torrado virus Kra isolate [16] was used as the virus source for plant inoculation.

### Cloning of the coding sequence of the putative 11K domain

All primers used in this study are listed in Table 1. Plants were inoculated with ToTV, and from those showing disease symptoms, total RNA was isolated using TRI Reagent (Life Technologies) as described previously [19]. Up to 2 µg of the RNA was reverse transcribed using 100 ng of random hexamers and 200 U of RevertAid Reverse Transcriptase (Thermo Scientific). The cDNA was used for polymerase chain reaction (PCR) with primers flanking the sequence between the first ATG codon from ToTV RNA1 (the 5UTR/PrCo_F primer) and the first codon of the protease cofactor motif (the 5UTR/PrCo_R primer) within the same RNA1. After agarose gel electrophoresis (1 % agarose in TBE buffer), DNA fragments were extracted from the gel and cloned into two expression vectors: pBIN61 and pgR107 [20]. Briefly, the vectors were digested with *Sma*I and treated with alkaline phosphatase (Thermo Scientific). One hundred nanograms of the re-purified *Sma*I-digested pBIN61 was used for recombination-based cloning with 300 ng of PCR-amplified 11K sequence using an In-Fusion cloning Kit (Clontech).

**Table 1:** Oligonucleotide primers used in this study

Primer ID	Primer sequence (5′→3′)	Primer application
5UTR/PrCo_F	TCTAGAGGATCCCCC**ATG**TCTTTTTCCAAGATGTTC	Cloning and expression of the ToTV 11K coding sequence. Start codon is in bold, stop codon is in italics. The 15-nucleotide underlined sequence was used for recombination-based cloning with pBIN61
5UTR/PrCo_R	GAATTCGAGCTCCCC*TCA*ATCACAAATTGATTTGTAC	
qsGFP1	CACATGAAGCAGCACGACTT	Real-time RT-PCR, quantitation of GFP mRNA
qsGFP2	TCCTTGAAGTCGATGCCCTT	
PVX1	GGATAGGAGTGGAACAATGA	Real-time RT-PCR, quantitation of PVX RNA-dependent RNA polymerase gene
PVX2	CAATTTCTCTCAATGCCTTC	
pgR107_CPF	CGCAACTCCTGCCACAGCTTCA	Real-time RT-PCR, quantitation of PVX coat protein gene
pgR107_CPR	GTCCCAAGCAGCCTGTGCCATA	
pgr107_25KF	ACCGTGCATACACTCGGTGTCC	Real-time RT-PCR, quantitation of PVX 25K gene
pgr107_25KR	CGAAATCGAAGCCACAGCCAGC	
NbActF	GTGAAGGAGAAGTTGGCTTAC	Real-time RT-PCR raw data normalisation, quantitation of *N. benthamiana* actin mRNA [17]
NbAct2	CTTCTGGGCAGCGGAATCTC	
NbEF1aF	CACCATTGATATTGCCTTGTG	Real-time RT-PCR raw data normalisation, quantitation of *N. benthamiana* EF1α mRNA [18]
NbEF1aR	GTTCTTGATAAAGTCCCTGTG	

The *Sma*I-digested pgR107 vector was ligated to the PCR-amplified 11K using T4 DNA ligase (Thermo Scientific). Both the recombination mixture and the ligation mixture were used to transform competent cells of *E. coli* strain TOP10 (Life Technologies). The recombined plasmid was extracted, its sequence was verified, and it was used for transformation of *A. tumefaciens* strains C58C1 (pBIN61-based constructs) or GV3101 (pgR107-based constructs).

### Local and systemic protein expression in *N. benthamiana*

A suspension of *A. tumefaciens* carrying either the pBIN61- or pgR107-based expression vector were grown at 28 °C for 24-48 h in LB medium with kanamycin and tetracycline. The bacteria were harvested and suspended in infiltration buffer (10 mM MES, pH 5.8, 0.5 μM acetosyringone, and 10 mM MgCl_2_) and kept at room temperature for at least 2 h. The mixture was adjusted to an OD_600_ of 1.0 and used to infiltrate two leaves of 6-week-old *N. benthamiana* seedlings, which were subsequently maintained under greenhouse conditions at 24-25 °C. Local protein expression was done using pBIN61-derived constructs, whereas both local and systemic expression of 11K was carried out using pgR107-11K (the PVX-based expression vector). For each infiltration, at least five plants were used, and the entire experiment was performed three times.

### In-patch GFP silencing assay

For the GFP-based silencing patch co-infiltration assay, the suspension of *A. tumefaciens* transformed with pBIN61-GFP (OD_600_ 0.1) was mixed with *Agrobacterium* (OD_600_ = 1.0) transformed with either pBIN61-11K, pBIN61-p19 or pBIN61 (empty vector) and co-infiltrated into leaves of *N. benthamiana*. Plants were maintained under greenhouse conditions at 24-25 °C. GFP fluorescence was excited using a hand-held UV lamp (UV Tech) and monitored 72 h after infiltration. Photographs were taken using a Nikon D5100 camera.

### RNA and protein analysis

Total RNA was extracted as described previously [19]. For reverse transcription (RT) quantitative real-time PCR (RT-qPCR), 1 μg of total RNA was first treated with DNase (Thermo Scientific) and then subjected to cDNA synthesis. The resulting cDNA was used for RT-qPCR with the appropriate primers (Table 1) and iTaq™ universal SYBR® Green Supermix (Bio-Rad). The raw Ct data were normalised against actin or EF1α mRNA.

Total soluble proteins were isolated from infiltrated leaf patches using extraction buffer (1 M Tris-HCl, pH 7.5, and 20 % glycerol). The homogenate was centrifuged (16,000 × *g*, 30 min, 4 °C) to remove cell debris, and the protein concentration was measured using a Bio-Rad Protein Assay (Bio-Rad). An equal amount (50 μg) of the proteins was subjected to sodium dodecyl sulfate polyacrylamide gel electrophoresis (SDS-PAGE). After electrophoresis, proteins were electroblotted onto a PVDF filter (Roche), and GFP was probed using primary monoclonal antibody (anti-GFP monoclonal antibody, Thermo Scientific). The GFP protein was visualised using Western Blue^®^ Stabilized Substrate for Alkaline Phosphatase (Promega).

### Bioinformatic analysis

Protein secondary structure was predicted using the GeneSilico Metaserver [21], and amino acid sequence alignments were done using ClustalW [22]. Sequence identities and similarities were calculated using BioEdit [23]. Protein sequence variability was assessed using the Protein Variability Server [24].

## Results

### The ToTV 11K domain induces an HR-like phenotype in *N. benthamiana*

**Fig. 2:**
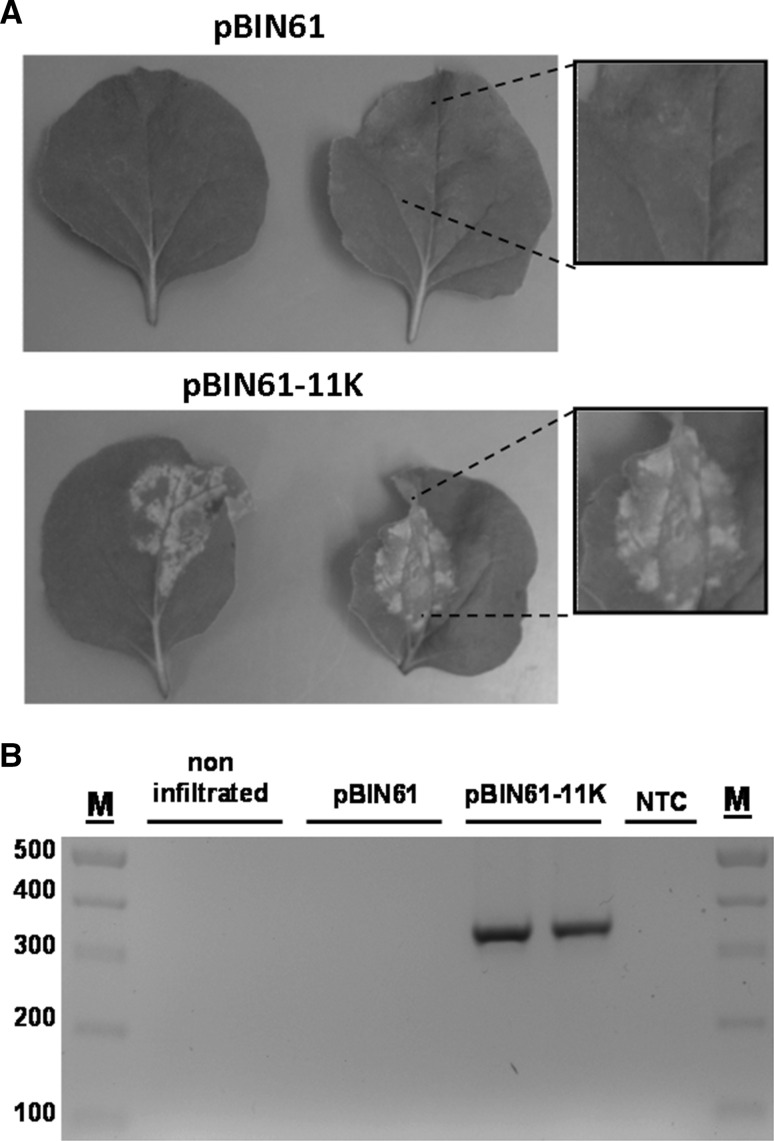
The 11K domain induces a hypersensitive response (HR)-like reaction in *Nicotiana benthamiana*. **A.** Leaves of *N. benthamiana* infiltrated with *Agrobacterium tumefaciens* carrying pBIN61 (upper panel) or pBIN61-11K vectors (lower panel). **B.** Detection of the 11K coding sequence in *N. benthamiana.* RNA was isolated from infiltrated leaves and used for RT-PCR with 11K-specific primers. The amplicon of *ca.* 350 nt was obtained only from plants infiltrated with pBIN61-11K. M, DNA mass ruler; NTC, no template control

To investigate the biological function of the 11K domain, which is composed of 105 amino acid residues upstream of the protease cofactor (ProCo) motif encoded on ToTV RNA1 (Fig. 1B), the coding region between the 5’UTR and the ProCo motif was amplified by RT-PCR and was inserted into the pBIN61 vector. As shown in Fig. 2A, when this construct was used to infiltrate *N. benthamiana* plants, an HR-like reaction was induced 72 hours post-infiltration (hpi) to produce pBIN61-11K. In comparison, patches infiltrated with the empty pBIN61 vector did not develop an HR-like phenotype. To determine whether the 11K RNA was transcribed in leaf patches infiltrated with pBIN61-11K, total RNA was extracted from the infiltrated leaves, treated with DNase and used for RT-PCR. As expected, a *ca.* 350-nt 11K-specific amplicon was obtained only when RNA template extracted from HR expressing leaves was used. Neither the RNA isolated from non-infiltrated plants nor RNA extracted from patches infiltrated with pBIN61 gave the expected amplification product (Fig. 2B). This demonstrates that the 11K domain ectopically expressed in *N. benthamiana* induces a cell-death-like reaction in the host.

### The 11K peptide enhances the pathogenicity of a chimeric PVX

Next, we investigated the effect of the ToTV 11K domain on the pathogenicity of a mild virus. For this purpose, the 11K domain was expressed in *N. benthamiana* using a PVX-based expression vector.

**Fig. 3:**
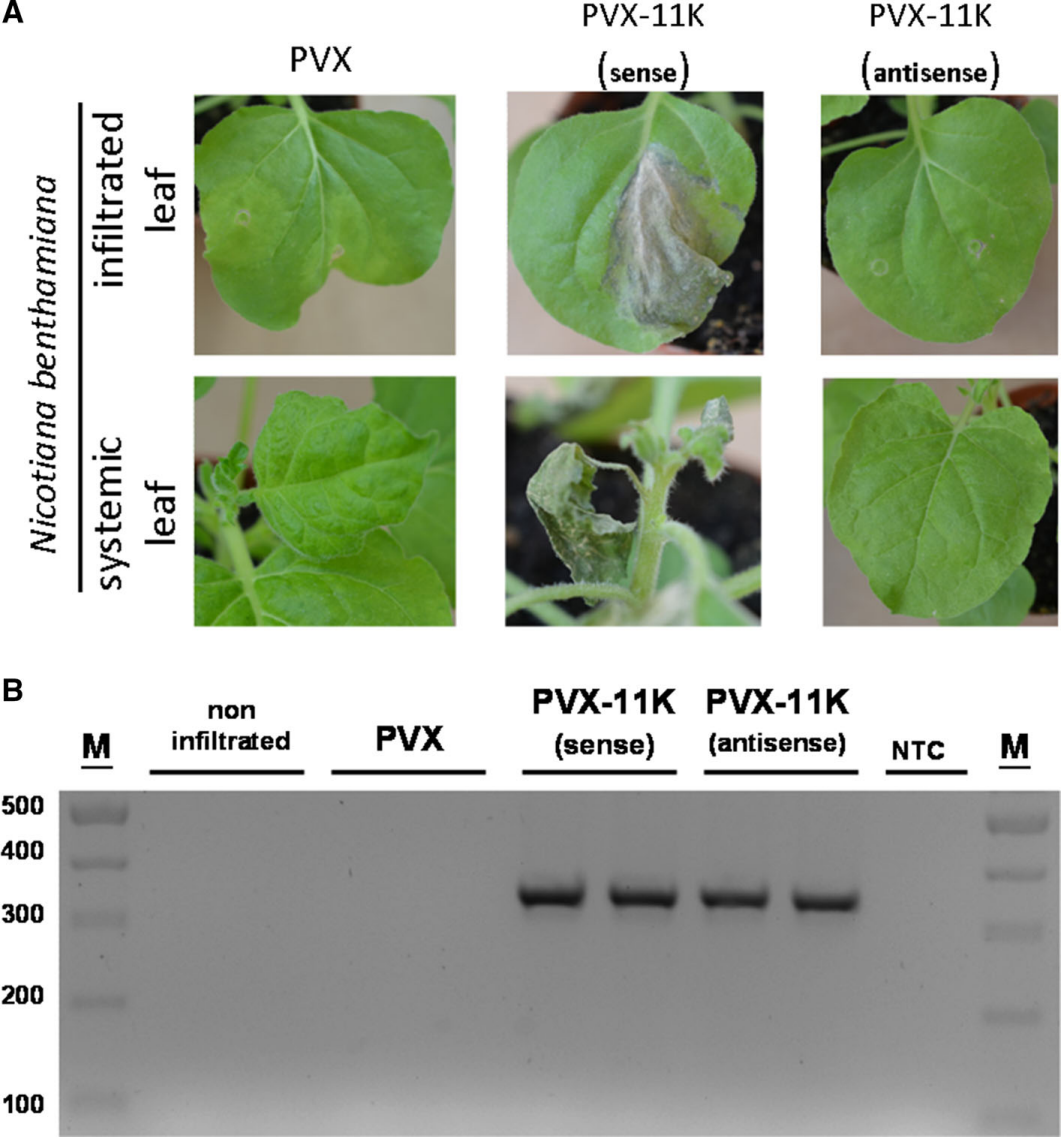
The 11K domain enhances virulence of a chimeric potato virus X (PVX) in *Nicotiana benthamiana*. **A.** *N. benthamiana* plants infiltrated with *Agrobacterium tumefaciens* carrying an infectious clone of PVX or PVX-11K with the 11K coding sequence in the sense or antisense orientation. **B.** RT-PCR-based detection of 11K transcripts in systemically infected leaves of *N. benthamiana*. The *ca.* 350-nt amplicon was obtained only from plants infected with PVX-11K. M, DNA mass ruler; NTC, no template control

PVX expressed from the pgR107 plasmid induced mild disease symptoms in *N. benthamiana*, and in emerging leaves of the plants infected with pgR107, only a mild mosaic was observed (Fig. 3A). However, leaves infiltrated with pgR107-11K developed a cell-death phenotype that was already visible at 3 dpi. Symptoms were stronger than those displayed by plants expressing the 11K domain from pBIN61-11K. Such a phenotype was not observed in leaves infiltrated with either pgR107 or pgR107-11Kas (Fig. 3A).

In the course of time, PVX infection expanded toward new leaves. PVX alone induced a mild disease phenotype in upper non-infiltrated leaves. Similar symptoms were observed in plants infected with PVX-11Kas. To assess whether the plants were actually infected with PVX or PVX-11Kas, total RNA was extracted from upper non-infiltrated leaves and subjected to RT-PCR with 11K-specific primers. As expected, the ca. 350-nt amplicon was obtained from RNA isolated from PVX-11K- and PVX-11Kas-infected plants (Fig. 3B). However, only in plants infected with PVX-11K was strong systemic necrosis observed. Necrosis started to develop around the veins and proceeded to the entire leaf. The reaction was so strong that plants could not develop new apical leaves and thus remained stunted (Fig. 3A).

**Fig. 4:**
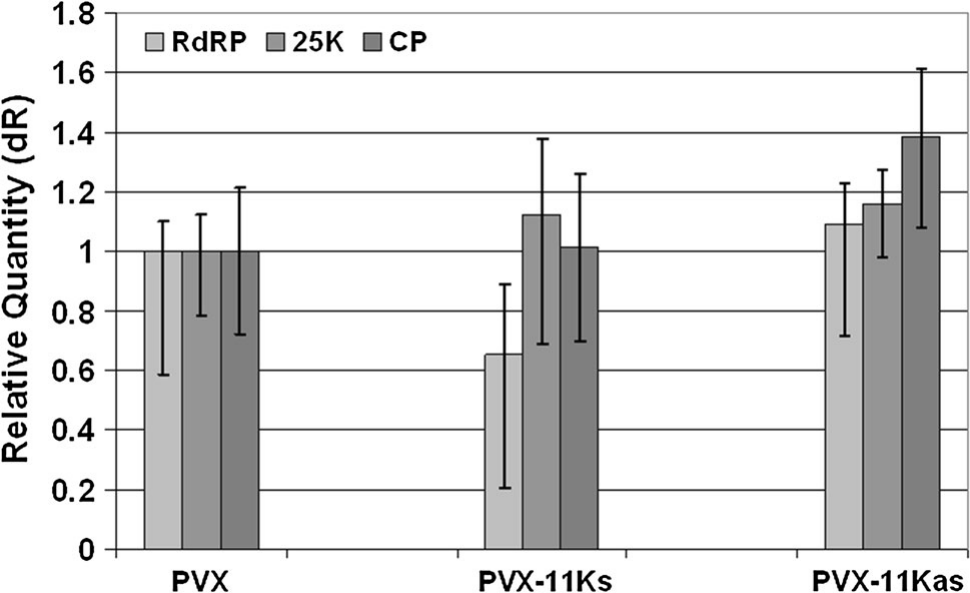
Comparative analysis of potato virus X RNA accumulation in *Nicotiana benthamiana* plants infected with PVX, PVX-11K or PVX-11Kas. The relative accumulation of three PVX open reading frames (RdRp, 25K and CP) was analysed by qRT-PCR

Next, we investigated whether the severe symptoms induced in PVX-11K-infected *N. benthamiana* were due to enhanced replication and therefore to an increase in accumulation of PVX-11K in infected tissues. For this purpose, total RNA was extracted from upper non-infiltrated leaves and used for RT-qPCR. The expression of three PVX genes (RdRP, 25K, and CP) was measured and normalized against the expression level of actin or *EF1α* genes. The results indicated that the levels of accumulated genomic (the RdRP gene) and subgenomic (25K and CP genes) PVX RNAs were comparable in plants infected with PVX, PVX-11K and PVX-11Kas (Fig. 4). In detail, the level of RdRP RNA decreased *ca.* 1.6-fold in plants infected with PVX-11K, whereas it increased slightly (*ca*. 1.2-fold) in those infected with PVX-11Kas. The expression level of 25K did not change considerably in PVX-11K-infected plants, but CP gene expression increased 1.4-fold in plants infected with PVX-11Kas, and it did not change in plants infected with PVX-11K (Fig. 4). This suggests that the severe disease symptoms observed in PVX-11K infection were not caused by a dramatic increase in PVX RNA expression but instead were induced by the biologically active 11K domain.

### The 11K domain does not suppress PTGS in *N. benthamiana*

**Fig. 5:**
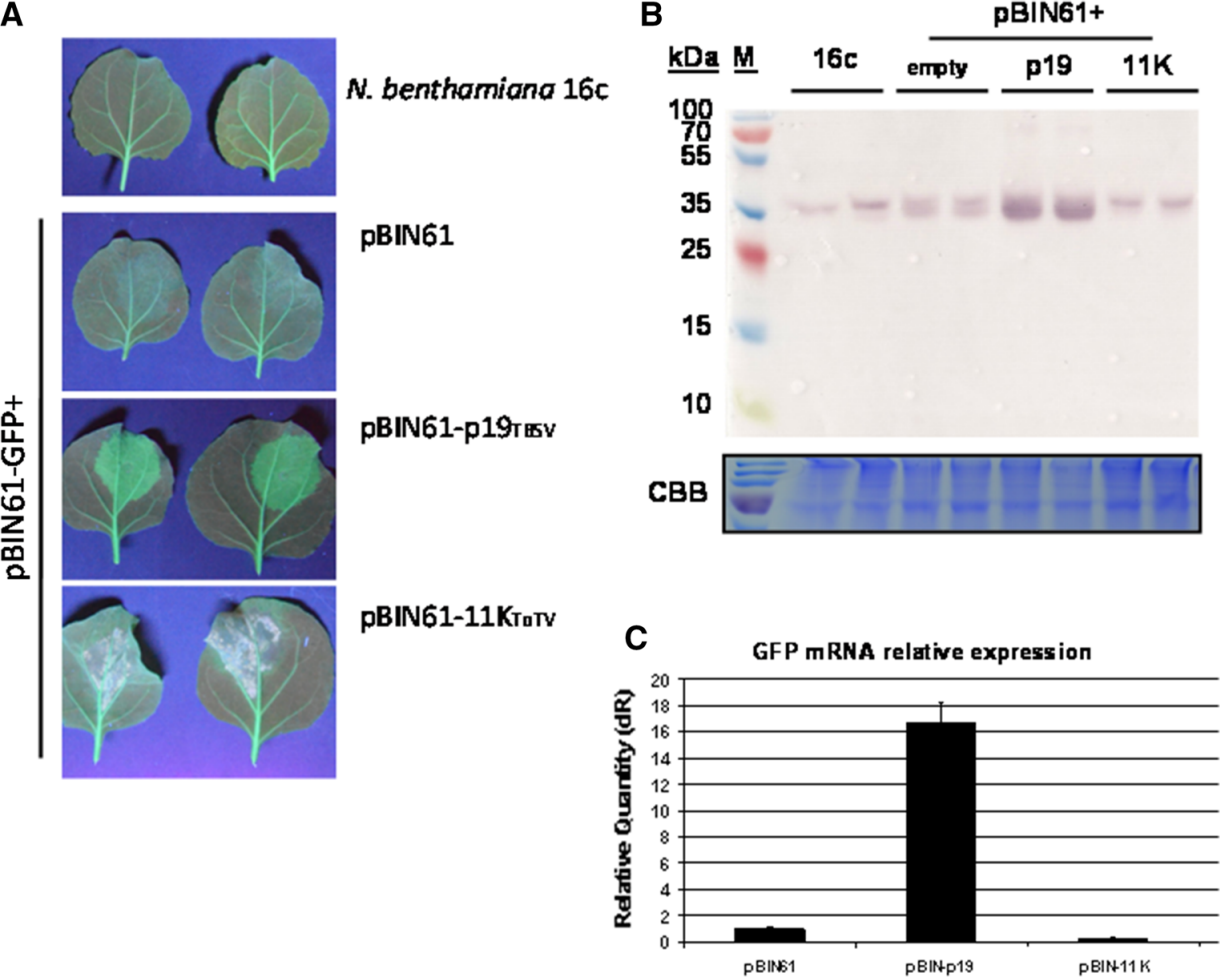
Analysis of posttranscriptional gene silencing (PTGS) suppressor activity of the ToTV 11K domain. **A.** Leaves of *Nicotiana benthamiana* 16c infiltrated with mixtures of *Agrobacterium tumefaciens* cultures carrying GFP (pBIN61-GFP) and either an empty vector (pBIN61), the p19 PTGS suppressor from tomato bushy stunt virus (pBIN61-p19) or the ToTV 11K domain (pBIN61-11K). **B.** Western blot analysis of GFP accumulation in infiltrated patches with p19 or 11K expression. **C.** Accumulation of GFP mRNA expressed together with the tomato bushy stunt virus p19 or the ToTV 11K domain

Thomas et al. [25] reported previously that the P38 protein (coat protein) from turnip crinkle virus induces severe necrosis when ectopically expressed from a PVX vector. In the same study, the authors showed a possible role of P38 as a suppressor of RNA silencing. To verify the hypothesis that the disease symptoms induced in *N. benthamiana* upon 11K expression were a consequence of suppression of PTGS pathways, a co-infiltration silencing assay was performed. Transient expression of GFP in *N. benthamiana* proceeded until 3 dpi and then decreased as a result of PTGS. However, in the presence of a PTGS suppressor, expression of the reporter gene was maintained at a high level. Indeed, at 3 dpi, strong GFP fluorescence was observed in leaf patches infiltrated with pBIN61-GFP together with pBIN61-p19 (a silencing suppressor protein of tomato bushy stunt virus [26]) (Fig. 5A). In comparison, GFP fluorescence was hardly observed in leaves infiltrated with pBIN61. Interestingly, in leaves co-expressing GFP and 11K, a brightening was observed (Fig. 5A). To determine whether the effect was caused by enhanced expression of GFP in infiltrated leaves, total soluble protein extracts were prepared from the GFP-expressing patches and were subjected to SDS-PAGE followed by western blot analysis with GFP-specific monoclonal antibodies. High GFP expression was confirmed in leaves co-infiltrated with pBIN61-GFP and pBIN61-p19 (Fig. 5B). However, in leaves expressing GFP together with pBIN61 or pBIN61-11K, the level of GFP was substantially lower. This suggests that the brightening in leaves infiltrated with pBIN61-11K was not due to increased GFP expression. This was also confirmed by comparing GFP mRNA levels. Relative RT-qPCR analysis showed that GFP mRNA accumulated to the highest level only when co-expressed with the p19 silencing suppressor (Fig. 5C).

### The predicted secondary structure of the ToTV 11K domain is conserved in the corresponding regions of tomato-infecting torradoviruses

**Fig. 6:**
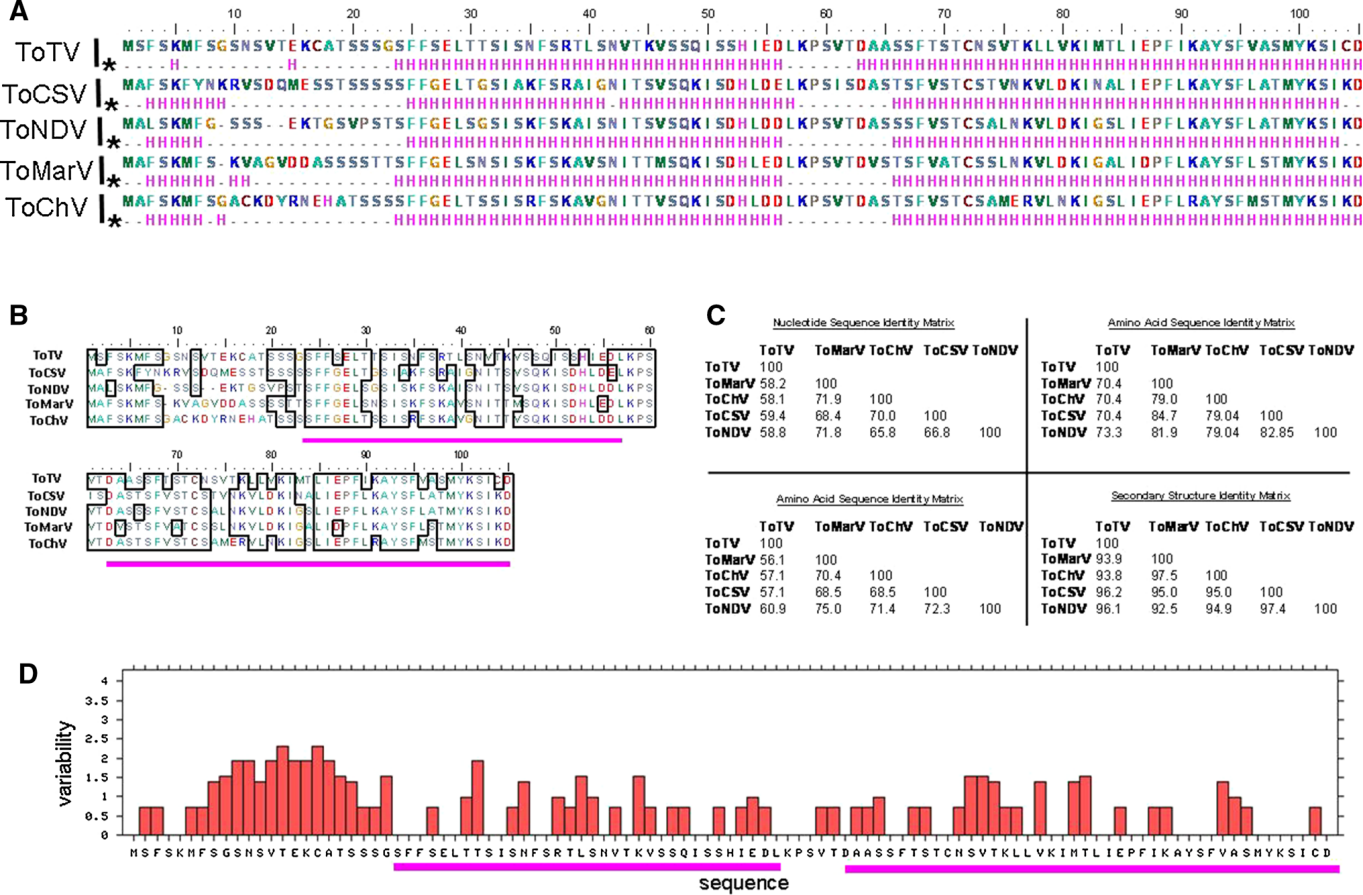
Sequence comparison of the 11K domains from tomato-infecting torradoviruses. **A.** Amino acid sequence alignment of the 11K domain. Primary structure and predicted helical domains (the rows marked with asterisks). H, amino acids forming α-helices. **B.** Sequence alignment of the corresponding 11K domains of tomato-infecting torradoviruses. Similar amino acids are outlined with black frames. Pink bars indicate predicted helices. **C.** Nucleotide, amino acid, and predicted helical domain similarity matrices of the 11K domains of tomato-infecting torradoviruses. **D.** Sequence variability plot of the 11K domain of tomato-infecting torradoviruses. The calculation was made according to Shannon’s method [24]. A score (y-axis) of 0 indicates conserved amino acids. A score higher than 0 indicate variable amino acid positions. The pink bars indicate the locations of the two predicted helical motifs. ToTV, tomato torrado virus; ToMarV, tomato marchitez virus; ToCSV, tomato chocolate spot virus; ToChV, tomato chocolate virus; ToNDV, tomato necrotic dwarft virus (color figure online)

The consensus prediction of putative secondary structure motifs in the 11K domain (based on eighteen predictors implemented in the GeneSilico Metaserver) indicated the potential to form two α-helical motifs. Importantly, no β-sheets were predicted to be formed within the 11K domain. Moreover, the predicted helical motifs consisted of two separate domains: helix 1 between amino acids 24 and 56, and helix 2 between amino acids 63 and 105 (Fig. 6A). The same analysis was performed for amino acid sequences of the corresponding regions of tomato marchitez virus, tomato necrotic dwarf virus, tomato chocolate virus and tomato chocolate spot virus. The two predicted helical motifs were found in all of the aforementioned viruses, at almost the same positions in their corresponding 11K sequences (Fig. 6A). Interestingly, those two predicted helices were composed of amino acids with similar properties (Fig. 6B), and although the RNA sequence differs substantially between the analogous regions of other torradoviruses, the predicted secondary structures in their 11K domains are highly conserved (Fig. 6B and C). Moreover, the proposed helix-building amino acids of the 11K domain had the lowest variability score (Fig. 6D). This confirmed that although the two helices in the 11K domain are sequence-defined, the domains have a conserved structure rather than a conserved sequence.

## Discussion

Tomato torrado virus is an emerging pathogen of tomato, in which it induces severe systemic necrosis of leaves. Moreover, ToTV can infect *N. benthamiana*, inducing chlorosis and leaf malformations. Importantly, necrosis of locally or systemically infected leaves is not observed in ToTV-infected *N. benthamiana*. We previously showed that the host dependence of ToTV is determined at least in part by the ToTV movement protein (3A) [8]. In the present study, we show that a short domain of the ToTV RNA1 polyprotein, named the 11K domain, can mediate an HR-like reaction in *N. benthamiana*.

Initially, we showed that the 11K domain can trigger a local HR-like reaction when transiently expressed in *N. benthamiana*. Plants infected with PVX-11K developed severe systemic necrosis leading to death of plant shoots. However, this effect was not observed in tomato cv. Beta Lux (data not shown). A similar necrotic reaction on *N. benthamiana* was observed by Thomas et al. [25]. In their preliminary studies on the biological functions of the turnip crinkle virus (TCV) coat protein (P38), these authors showed that expression of P38 from a PVX vector led to induction of necrosis within the infiltrated leaves followed by plant shoot death. The same authors showed that the P38-trigerred necrosis was associated with increased accumulation of the PVX-P38 chimeric virus. Similarly, Aguilar et al. [27] described a local HR-like necrosis response induced upon expression of chimeric PVX in *N. benthamiana*. The authors used PVX with an inserted coding sequence of an RNA silencing suppressor: HCPro from plum pox virus or p19 from tomato bushy stunt virus. Expression of both proteins resulted, on one hand, in induction of an HR-like response in infiltrated leaves, and on the other hand, to increased accumulation of PVX subgenomic RNAs but not genomic RNA. These observations were characteristic for PVX expressing PTGS suppressors from other viruses. However, such an effect was not observed for the 11K domain in our study. Accumulation of the necrosis-inducing PVX-11K in systemic leaves was comparable in symptomless PVX and PVX-11Kas.

Next, it was essential to determine whether the necrosis-inducing 11K was also able to suppress PTGS in transgenic *N. benthamiana*. Although some leaf brightening was observed in *N. benthamiana* leaves co-expressing GFP and the 11K domain, we could not associate this effect with increased GFP accumulation. Western blot analysis and RT-qPCR confirmed the absence of PTGS suppressor activity of the 11K domain.

The 11K domain is comprised of 105 amino acid residues. *In silico* prediction indicated that at least 80 % of the amino acid residues might be involved in forming the two long alpha helices. These two helices (helix1 and helix2) are located at the same positions in the region corresponding to the 11K domain of all tomato-infecting torradoviruses. This suggests that the conserved structure of the 11K domain and the effect induced upon its expression in *N. benthamiana* (but not in tomato) might reflect the involvement of the 11K domain in host-specific plant-virus interactions. We previously pointed out that the host specificity of ToTV might be mediated by its movement protein (3A) by showing that ToTV with an F210L mutation in the RNA2 polyprotein was not able to infect tomato cv. Beta Lux but remained infectious to *N. benthamiana* [8]. Moreover, single amino acid substitutions in Vp26 (one of the three ToTV coat protein subunits) diminished the ability of ToTV to infect tomato [Wieczorek et al. submitted]. The same substitutions had no effect on ToTV infectivity in *N. benthamiana*. Therefore, it cannot be ruled out that the biological activity of the ToTV 11K domain acts in a host-specific manner.

It was previously shown that helical motifs in different viral proteins might be involved in induction of disease-like symptoms in *N. benthamiana*. Mochizuki et al. [28] showed that necrotic spots appeared on *N. benthamiana* upon expression of the replication protein p29 of melon necrotic spot virus. By creating several deletion variants of p29, the authors pointed out a potential hydrophobic α-helix in transmembrane domain 2 (TMD2) as essential for inducing necrotic spots in *N. benthamiana*. Hashimoto et al. [29] showed that a putative amphipathic helix in the helicase protein (Hel) of radish mosaic virus is responsible for membrane modification leading to cell death and necrotic symptoms in *N. benthamiana*.

Little is known about ToTV replication, and likewise, there are no data describing whether ToTV replication complexes form in association with cell membranes. Membrane-associated viral proteins might contain transmembrane motifs or amphipathic helices that are likely to modify host membranes. Hashimoto et al. [29] tested whether other amphipathic helix-containing viral proteins of two other members of the family *Secoviridae* would induce a cell death phenotype in *N. benthamiana*. Indeed, amphipathic helix-containing Hel proteins of cowpea mosaic virus and tobacco ringspot virus induced cell death when expressed in *N. benthamiana*, although the viruses do not induce systemic necrosis in the host. Consistent with this, it should be kept in mind that naturally infecting ToTV does not induce systemic necrosis in *N. benthamiana*. Komatsu et al. [30] showed that the necrosis-inducing activity of Plantago asiatica mosaic virus RNA-dependent RNA polymerase triggered systemic necrosis in *N. benthamiana* in a dose-dependent manner and was modulated by its helicase domain. In this light, the molecular mode of action of the necrosis-inducing ToTV 11K domain might be more complex. Importantly, it should be taken into account that not all amphipathic helices are directly responsible for induction of a cell death phenotype [31]. Therefore, in the case of the ToTV 11K domain, its putative amphipathic helix and other potential necrosis-modulating factors have to be more precisely analysed in the context of ToTV infectivity in *N. benthamiana*.
